# Corneal ulcerative disease in dogs under primary veterinary care in England: epidemiology and clinical management

**DOI:** 10.1186/s40575-017-0045-5

**Published:** 2017-06-15

**Authors:** Dan G. O’Neill, Monica M. Lee, Dave C. Brodbelt, David B. Church, Rick F. Sanchez

**Affiliations:** 1 Pathobiology and Population Sciences, The Royal Veterinary College, Hawkshead Lane, North Mymms, Hatfield, Herts AL9 7TA UK; 2 The Royal Veterinary College, Hawkshead Lane, North Mymms, Hatfield, Herts AL9 7TA UK; 3 Clinical Science and Services, The Royal Veterinary College, Hawkshead Lane, North Mymms, Hatfield, Herts AL9 7TA UK; 4 Speciaslistische Dierenkliniek Utrecht, Middenwetering 19, Utrecht, Netherlands

**Keywords:** General practice, First opinion, VetCompass, Ulcerative keratitis, Corneal ulceration, Brachycephalic, Spaniel

## Abstract

**Background:**

Corneal ulcerative disease (CUD) has the potential to adversely affect animal welfare by interfering with vision and causing pain. The study aimed to investigate for the first time the prevalence, breed-based risk factors and clinical management of CUD in the general population of dogs under primary veterinary care in England.

**Results:**

Of 104,233 dogs attending 110 clinics participating within the VetCompass Programme from January 1^st^ to December 31^st^ 2013, there were 834 confirmed CUD cases (prevalence: 0.80%, 95% confidence interval (CI) 0.75–0.86). Breeds with the highest prevalence included Pug (5.42% of the breed affected), Boxer (4.98%), Shih Tzu (3.45%), Cavalier King Charles Spaniel (2.49%) and Bulldog (2.41%). Purebred dogs had 2.23 times the odds (95% CI 1.84–2.87, *P* < 0.001) of CUD compared with crossbreds. Brachycephalic types had 11.18 (95% CI 8.72–14.32, *P* < 0.001) and spaniel types had 3.13 (95% CI 2.38–4.12, *P* < 0.001) times the odds for CUD compared with crossbreds. Pain was recorded in 385 (46.2%) cases and analgesia was used in 455 (54.6%) of dogs. Overall, 62 (7.4%) cases were referred for advanced management and CUD contributed to the euthanasia decision for 10 dogs.

**Conclusions:**

Breeds such as the Pug and Boxer, and conformational types such as brachycephalic and spaniels, demonstrated predisposition to CUD in the general canine population. These results suggest that breeding focus on periocular conformation in predisposed breeds should be considered in order to reduce corneal disease.

## Plain English summary

The cornea is the outermost layer of the eye and it must remain transparent to support vision. Ulcerated corneas can lose transparency and become particularly painful. Understanding which breeds and conformational types of dog in primary-care practices are commonly affected by corneal disease could assist in developing preventive measures such as breeding strategies or improved eye care and guide veterinary surgeons in primary-care practice. Information collected directly from the clinical records of first opinion veterinary practices by the VetCompass Programme can provide reliable health data on the general population of dogs in the UK, and was used here for the first time to explore the clinical picture associated with ‘corneal ulcerative disease.

Of 104,233 dogs attending 110 primary-care practices in England, there were 834 dogs with corneal ulceration (0.80% overall). The breeds with the highest prevalence were the Pug (5.42% of the breed affected), Boxer (4.98%), Shih Tzu (3.45%), Cavalier King Charles Spaniel (2.49%) and Bulldog (2.41%). Pain associated with the disease was recorded in 46.2% of cases, at least one painkiller was dispensed or administered in 54.6, and 17.0% underwent surgery.

Compared with crossbred dogs, the Pug was 19 times as likely and the Boxer was 12 times as likely to have corneal ulceration. In comparison with crossbred dogs, flat-faced dogs (brachycephalics) were 11 times as likely and spaniel types were 3 times as likely to have corneal ulceration.

These results demonstrate for the first time by using large numbers of primary-care practice cases that brachycephalic breeds have a predisposition for corneal ulcerative disease. The strong breed predispositions suggests the opportunity to improve animal welfare by focusing on breed-based preventive strategies.

## Background

The outermost layer of the cornea is a richly innervated epithelium that protects the underlying stroma and supports the endothelium in maintaining a dehydrated stroma, which is essential for corneal transparency [1]. Epithelial defects that affect the basement membrane and expose the corneal stroma are known as corneal ulcers [2]. Corneal ulcerative disease (CUD) can affect animal welfare by the development of pain, reflex uveitis, perforation and even loss of the eye and possibly also by temporarily or permanently interfering with vision [3, 4].

The majority of published epidemiological studies on canine CUD are based on referral populations and therefore are biased towards selection of more complicated cases and towards outcomes that are heavily influenced by the post-referral skill-sets and equipment [5–10]. Although findings from referral studies may be useful for referral practitioners, these results are likely to be poorly generalisable to general primary-care caseloads and have limited applicability for quantifying disorder levels in broader dog populations [11]. It is therefore important for ophthalmologists, general clinicians and welfare scientists to access clinical research results from primary-care practice in order to offer informed advice relevant to the general primary-care CUD caseloads even if, and especially where, the standards of care differ from the norms in referral practice [12]. Welfare scientists can benefit from access to primary-care prevalence data that can assist with disorder prioritisation across all dogs and from access to breed risk factor data that can assist with focused prioritisation within individual breeds [12–14].

Corneal ulcerative disease describes a broad clinical presentation resulting from a variety of underlying causes and predispositions that may or may not be formally identified during the clinical work-up [15]. Primary causes of CUD in dogs include spontaneous chronic corneal ulceration (SCCED) [5, 10, 16] and canine herpes virus-1 [17]. Multiple secondary causes are reported, including entropion [18, 19], ectopic cilia [8, 20], primary and secondary forms of keratoconjunctivitis sicca (KCS) [7, 21], corneal degeneration [22], traumatic events [23–25], corneal overexposure related to general anesthesia [26], facial nerve paralysis [27] and orbital diseases [28, 29]. Many of these factors have also been associated with certain breed phenotypes [30] and some smaller studies based on referral populations of less than 250 cases have reported increased CUD prevalence in breed-types such as brachycephalic [7, 31, 32] or spaniel types [7, 33]. However, CUD has not been conclusively associated with particular breed-types or conformational signalment in the general dog population mainly because general population studies with large enough numbers of cases have been lacking to date. The present study aimed to investigate the prevalence, risk factors and clinical management for CUD as diagnosed by veterinary practitioners in the general population of dogs attending primary-care practices enrolled in the UK VetCompass Programme in order to give a picture of the occurrence, diagnostics and outcomes of the condition in this setting. It was hypothesized that brachycephalic and spaniel types have higher odds of CUD than crossbred dogs.

## Methods

The VetCompass Programme collates de-identified electronic patient record (EPR) data from primary-care veterinary practices in the UK for epidemiological research [34]. Collaborating practices can record summary diagnosis terms from an embedded VeNom Code [35] list during episodes of care. VetCompass collects information fields that include species, breed, date of birth, sex, neuter status, insurance status and bodyweight, and clinical information from free-form text clinical notes and summary diagnosis terms (VeNom codes), plus treatment and deceased status with relevant dates. The EPR data were extracted from practice management systems using integrated clinical queries and uploaded to a secure VetCompass structured query language database [36].

A cross-sectional analysis using cohort clinical data of dogs attending VetCompass practices was used to estimate the prevalence, risk factors and clinical management for CUD [37]. The sampling frame for the current study included dogs under veterinary care within the VetCompass database from January 1^st^ 2013 to December 31^st^ 2013. Dogs ‘under veterinary care’ were defined as any dog that had either at least one EPR recorded from January 1^st^ to December 31^st^ 2013 or, alternatively, at least one EPR both before and after 2013. Sample size calculations estimated that 2,958 dogs of a specific type (e.g., brachycephalic or spaniel) and 11,832 crossbred dogs would be required to detect an odds ratio of 2.0 times or greater for CUD assuming a 0.5% prevalence of CUD in the crossbred dogs (4:1 ratio of crossbred:specific type, two-sided 95% confidence interval, 80% power) [38]. Ethical approval was granted by the RVC Ethics and Welfare Committee (reference number 2015/T94).

Case inclusion criteria required that a final diagnosis of CUD was recorded in the EPR and that corneal ulceration was present during the 2013 study period. Case-finding involved initial screening of all EPRs for candidate CUD cases by searching the clinical free-text field using search terms including *fluo* and *pos*, *fluo* and *+*, *corn* and *ulc*, *eyelid flap*, *TEF*, *keratect*, *descem*, and the VeNom term field using the search term *corneal ulcer*. The candidate cases were randomly ordered by the Microsoft Excel *RAND* function (Microsoft Office Excel 2007, Microsoft Corp.) and the clinical notes of each candidate were manually reviewed in detail to evaluate them for case inclusion. All dogs with confirmed CUD were grouped as *CUD cases* for the analysis, and all remaining study dogs were grouped as *non-cases*. Additional data were extracted on confirmed CUD cases including the date of first diagnosis, history of previous CUD events, laterality of eye/s affected, diagnostic tests used, whether pain was recorded in the clinical notes or not, types of medical and surgical therapy used, whether the animal was referred for advanced case management, CUD clinical status at the final record available, CUD recurrence in the same eye and whether CUD was associated with decisions to euthanase.

A *purebred* variable categorised all dogs of recognisable breeds as ‘purebred’ and the remaining dogs as ‘crossbred’ [39]. A *breed* variable included individual breeds with 10 or more CUD cases, a grouped category of all remaining purebreds and a general grouping of crossbred dogs. This approach was taken to allow focus on commonly affected breeds and to facilitate statistical power for the individual breed analyses [40]. A *spaniel-brachycephalic* variable grouped dogs into four categories: spaniel, brachycephalic, other purebreds or crossbred. Spaniel breeds were selected based on the inclusion of the word ‘spaniel’ in their name and included American Cocker, Brittany, Cavalier King Charles, Clumber, English Cocker, English Springer, English Toy, Field, Japanese (also known as Japanese Chin), King Charles, Picardy, Sussex, Tibetan, Welsh Cocker, Working Cocker, Water Spaniel and Welsh Springer. Brachycephalic breeds were selected based on breeds commonly included in studies on brachycephaly [41–44] and included American Bulldog, Boston Terrier, Boxer, English Bulldog, French Bulldog, Pekingese, Pug, Shih Tzu and Victorian Bulldog. A *Kennel Club breed group* variable classified breeds recognised by the UK Kennel Club into their relevant breed groups (gundog, hound, pastoral, terrier, toy, utility and working) and all remaining types were classified as non-Kennel Club recognised [45]. *A neuter* variable described the status of the dog (neutered or entire) recorded at the final EPR. An *insurance* variable described whether a dog was insured at any point during the study period. An *age* variable categorised age (years) into six groups (<3.0, 3.0–5.9, 6.0–8.9, 9.0–11.9, ≥ 12.0, not recorded). Age (years) was calculated for case animals at the date of diagnosis of the current corneal ulceration event and for non-case dogs at either July 1^st^, 2013 for dogs born before this date or at December 31^st^, 2013 otherwise. An *adult bodyweight* variable categorised adult bodyweight into six groups (0.0–9.9 kg, 10.0–19.9 kg, 20.0–29.9 kg, 30.0–39.9 kg, ≥ 40.0 kg, not available). Adult bodyweight described the maximum bodyweight recorded during the study period for dogs older than 9 months. A *bodyweight relative to breed mean* variable characterised the adult bodyweight of individual dogs as either below or equal/above the mean adult bodyweight for their breed and sex within the overall study population. This variable allowed the effect of adult bodyweight to be assessed *within* each breed/sex combination.

Following data checking and cleaning in Excel (Microsoft Office Excel 2013, Microsoft Corp.), analyses were conducted using Stata Version 13 (Stata Corporation). The 1-year period prevalence with 95% confidence intervals (CI) described the probability of CUD at any time during the 1-year 2013 study period. The CI estimates were derived from standard errors, based on approximation to the normal distribution [46]. Descriptive statistics characterised the purebred status, breed, Kennel Club breed group, spaniel-brachycephalic, sex, neuter status, insurance, age, adult bodyweight and bodyweight relative to breed mean for the case and non-case dogs. Clinical management regimes were reported and analysed for the CUD cases using the chi-square test to compare categorical variables and the Mann-Whitney *U* test to compare continuous variables [46].

Binary logistic regression modelling was used to evaluate univariable associations between risk factors (*purebred, breed, Kennel Club breed group, spaniel-brachycephalic, adult bodyweight, bodyweight relative to breed mean, age, sex, neuter* and *insurance*) and diagnosis of CUD. Because breed was a factor of primary interest for the study, *purebred, spaniel-brachycephalic* and *Kennel Club breed group* (variables that are highly collinear with breed) and *adult bodyweight* (a defining characteristic of individual breeds) were excluded from the initial breed multivariable modelling. Instead, each of these variables individually replaced the *breed* variable in the main final model in order to evaluate their effects after taking account of the other variables. Risk factors with liberal associations in univariable modelling (*P* < 0.2) were taken forward for multivariable evaluation. Model development used manual backwards stepwise elimination. Pair-wise interaction effects were evaluated for the final model variables and confounding effects from dropped variables were assessed by individual re-introduction to the final model. The area under the ROC curve was used to evaluate the quality of the model fit (non-random effect model) [47]. Statistical significance was set at *P* < 0.05.

## Results

The study population comprised 104,233 dogs attending 110 primary-care practices in England. Following manual review, 834 dogs met the CUD inclusion criteria giving an overall 1-year period prevalence of 0.80% (95% CI 0.75–0.86).

Individual breeds with the highest CUD prevalence included Pug (5.42% of the breed affected, 95% CI 4.11–7.00), Boxer (4.98%, 95% CI 3.89–6.26), Shih Tzu (3.45%, 95% CI 2.70–4.33), Cavalier King Charles Spaniel (2.49%, 95% CI 1.89–3.20) and Bulldog (2.41%, 95% CI 1.46–3.74) (Table 1). The CUD prevalence in the brachycephalic group was 3.76% (95% CI 3.30–4.25) and in the spaniel group was 1.31% (95% CI 1.10–1.56).

**Table 1:** Prevalence of corneal ulcerative disease in commonly affected dog breeds

Breed	No. cases	No. dogs in study	Prevalence %	95% CI^a^
Pug	55	1015	5.42	4.11–7.00
Boxer	69	1386	4.98	3.89–6.26
Shih Tzu	70	2031	3.45	2.70–4.33
Cavalier King Charles Spaniel	58	2332	2.49	1.89–3.20
Bulldog	19	787	2.41	1.46–3.74
King Charles Spaniel	11	496	2.22	1.11–3.93
Lhasa Apso	19	892	2.13	1.29–3.31
French Bulldog	12	642	1.87	0.97–3.24
West Highland White Terrier	28	2859	0.98	0.65–1.41
Staffordshire Bull Terrier	66	6863	0.96	0.74–1.22
Cocker Spaniel	37	4234	0.87	0.62–1.20
English Springer Spaniel	19	2210	0.86	0.52–1.34
Border Terrier	11	1327	0.83	0.41–1.48
Chihuahua	17	2223	0.76	0.45–1.22
Yorkshire Terrier	24	3354	0.72	0.46–1.06
Jack Russel Terrier	41	6699	0.61	0.44–0.83
Border Collie	12	2807	0.43	0.22–0.75
Crossbreed	88	23329	0.38	0.30–0.46
Labrador Retriever	24	9541	0.25	0.16–0.37
Other purebreds	154	29206	0.53	0.45–0.62
Overall total	834	104233	0.80	0.75–0.86

Prevalence of diagnosis of corneal ulcerative disease in commonly affected dog breeds attending primary-care veterinary practices in England

^a^CI confidence interval

Of the CUD cases with complete data available for that variable, 746 (89.5%) were purebred, 382 (45.8%) were female, 436 (78.0%) were neutered and 331 (63.5%) were insured. Dogs with CUD had a median adult bodyweight of 12.7 kg (IQR: 8.6–25.0, range: 1.6–96.5) and median age at diagnosis overall of 7.20 years (IQR: 3.50–9.80 range: 0.0–19.0). The most common breeds among the CUD cases were Shih Tzu (70/834 cases, 8.4% of all cases), Boxer (69/834, 8.3%), Staffordshire Bull Terrier (66/834, 7.9%), Cavalier King Charles Spaniel (58/834, 7.0%) and Pug (55/834, 6.6%) along with crossbred dogs (88/834, 10.6%) (Table 2). Of the non-case dogs with complete data on the variable, 80,109 (77.5%) were purebred, 49,180 (47.7%) were female, 46,050 (76.9%) were neutered and 31,840 (52.3%) were insured. The median adult bodyweight for non-cases was 17.6 (IQR: 9.4–29.0, range: 1.1–109.0) kg and the median age was 4.9 years (IQR: 2.1–8.5, range: 0.0–31.2). The most common breeds among the non-case dogs were Labrador Retriever (9,517, 9.2%), Staffordshire Bull Terrier (6,797, 6.6%), Jack Russell Terrier (6,658, 6.4%) and Cocker Spaniel (4,197, 4.1%) as well as 23,241 (22.5%) crossbreds (Table 2). Data completeness varied between the variables assessed: breed 100.0%, age 100.0%, sex 99.7%, bodyweight (aged > 9 months) 81.8%, insurance 58.9%, and neuter 58.0%.

**Table 2:** Descriptive and univariable logistic regression results

Variable	Category	Case No. (%)	Non-case No. (%)	Odds ratio	95% CI^a^	*P*-value
Purebred status	Crossbred	88 (10.55)	23243 (22.49)	Base		
	Purebred	746 (89.45)	80109 (77.51)	2.46	1.97–3.07	< 0.001
Common breeds	Crossbreed	88 (10.55)	23241 (22.48)	Base		
	Pug	55 (6.59)	960 (0.93)	15.13	10.74–21.32	< 0.001
	Boxer	69 (8.27)	1317 (1.27)	13.84	10.05–19.06	< 0.001
	Shih Tzu	70 (8.39)	1961 (1.90)	9.43	6.86–12.95	< 0.001
	Cavalier King Charles Spaniel	58 (6.95)	2274 (2.20)	6.74	4.82–9.41	< 0.001
	Bulldog	19 (2.28)	768 (0.74)	6.53	3.96–10.78	< 0.001
	King Charles Spaniel	11 (1.32)	485 (0.47)	5.99	3.18–11.28	< 0.001
	Lhasa Apso	19 (2.28)	873 (0.84)	5.75	3.48–9.48	< 0.001
	French Bulldog	12 (1.44)	630 (0.61)	5.03	2.74–9.24	< 0.001
	West Highland White Terrier	28 (3.36)	2831 (2.74)	2.61	1.70–4.00	< 0.001
	Staffordshire Bull Terrier	66 (7.91)	6797 (6.57)	2.56	1.86–3.53	< 0.001
	Cocker Spaniel	37 (4.44)	4197 (4.06)	2.33	1.58–3.42	< 0.001
	English Springer Spaniel	19 (2.28)	2191 (2.12)	2.29	1.39–3.77	0.001
	Border Terrier	11 (1.32)	1316 (1.27)	2.21	1.18–4.14	0.014
	Chihuahua	17 (2.04)	2206 (2.13)	2.04	1.21–3.43	0.008
	Yorkshire Terrier	24 (2.88)	3330 (3.22)	1.90	1.21–2.99	0.005
	Jack Russell Terrier	41 (4.92)	6658 (6.44)	1.63	1.12–2.36	0.010
	Border Collie	12 (1.44)	2795 (2.70)	1.13	0.62–2.08	0.684
	Labrador Retriever	24 (2.88)	9517 (9.20)	0.67	0.42–1.05	0.078
	Other purebreds	154 (18.47)	29052 (28.10)	1.40	1.08–1.82	0.012
Kennel Club Breed Groups	Breed not Kennel Club recognised	139 (16.67)	31185 (30.16)	Base		
	Toy	188 (22.54)	12413 (12.00)	3.40	2.73–4.24	< 0.001
	Utility	151 (18.11)	8681 (8.40)	3.90	3.10–4.92	< 0.001
	Terrier	120 (14.39)	13537 (13.09)	1.99	1.56–2.54	< 0.001
	Gundog	101 (12.11)	20551 (19.88)	1.10	0.85–1.43	0.456
	Hound	22 (2.64)	4525 (4.38)	1.09	0.69–1.71	0.706
	Pastoral	31 (3.72)	7467 (7.22)	0.93	0.63–1.38	0.721
	Working	82 (9.83)	5040 (4.87)	3.65	2.77–4.80	< 0.001
Spaniel-brachycephalic	Crossbred	88 (10.6)	23243 (22.5)	Base		
	Spaniel	130 (15.6)	9887 (9.6)	3.47	2.65–4.56	< 0.001
	Brachycephalic	239 (28.7)	6124 (5.9)	10.31	8.06–13.18	< 0.001
	Purebred other	377 (45.2)	64098 (62.0)	1.55	1.23–1.96	< 0.001
Adult (> 9 months) bodyweight (kg)	< 10.0	264 (31.65)	24731 (23.92)	1.63	1.28–2.08	< 0.001
	10.0–19.9	245 (29.38)	23047 (22.29)	1.59	1.25–2.03	< 0.001
	20.0–29.9	145 (17.39)	18302 (17.70)	1.20	0.92–1.56	0.186
	30.0–39.9	88 (10.55)	13301 (12.86)	Base		
	≥ 40.0	32 (3.84)	6564 (6.35)	0.70	0.46–1.07	0.097
	Not available	60 (7.19)	17454 (16.88)	0.58	0.43–0.79	0.001
Bodyweight relative to breed mean	Equal/Higher	389 (46.64)	40713 (39.37)	1.12	0.97–1.29	0.114
	Lower	372 (44.60)	43689 (42.25)	Base		
	Not recorded	73 (8.75)	18997 (18.37)	0.45	0.35–0.58	< 0.001
Age category (years) (pre-existing cases included as not-recorded)	< 3.0	188 (22.54)	33817 (32.71)	Base		
	3.0–5.9	136 (16.31)	26381 (25.51)	0.93	0.74–1.16	0.504
	6.0–8.9	232 (27.82)	20062 (19.40)	2.08	1.71–2.52	< 0.001
	9.0–11.9	172 (20.62)	13307 (12.87)	2.33	1.89–2.86	< 0.001
	≥ 12.0	106 (12.71)	9633 (9.32)	1.98	1.56–2.51	< 0.001
	Not recorded	0 (0.00)	199 (0.19)	~	~	~
Sex	Female	382 (45.80)	49180 (47.68)	Base		
	Male	452 (54.20)	53933 (52.29)	1.08	0.94–1.24	0.276
	Not recorded	0 (0.00)	26 (0.03)	~	~	~
Neuter status	Entire	123 (14.75)	13875 (13.42)	Base		
	Neutered	436 (52.28)	46050 (44.54)	1.07	0.87–1.31	0.521
	Not recorded	275 (32.97)	43474 (42.04)	0.71	0.58–0.88	0.002
Insurance	Non-insured	190 (22.78)	29062 (28.11)	Base		
	Insured	331 (39.69)	31840 (30.79)	1.59	1.33–1.90	< 0.001
	Not recorded	313 (37.53)	42497 (41.10)	1.13	0.94–1.35	0.197

Descriptive and univariable logistic regression results for risk factors associated with diagnosis of corneal ulcerative disease in dogs attending primary-care veterinary practices in England. Percentages shown in brackets

^a^CI confidence interval

Fluorescein staining was the most commonly used ancillary diagnostic aid, used in 774 (92.8%) cases. Schirmer tear test-1 (STT-1) strips were used in 198 (23.7%) cases, and bacteriology was used in 11 (1.3%) cases. There were 53 (6.4%) CUD cases diagnosed without the recorded use of any of these three tests. The laterality of the affected eye was recorded in 820 (98.3%) of cases. The left eye was affected in 390 (46.8%) cases, the right eye was affected in 382 (45.8%) cases and both eyes were affected in (5.8%) cases. Of the 834 confirmed CUD cases, 770 (92.3%) had no recorded history of a prior CUD event. Of the 64 cases with a history of prior CUD, the same eye was previously affected in 29 (45.5%), the contralateral eye was previously affected in 22 (34.4%) and both eyes were previously affected in 13 (20.3%).

Pain associated with CUD was recorded in the clinical notes of 385 (46.2%) cases. At least one analgesic agent was used in 455 (54.6%) of dogs. Overall, 576 (69.1%) of CUD cases had either pain recorded in their notes and/or received an analgesic agent. Of the 455 dogs receiving pain management, the most commonly used analgesic agents were non-steroidal anti-inflammatory drugs (*n* = 421, 92.5%), cycloplegics (36, 7.9%), opioids (25, 5.5%) and local anaesthetic (24, 5.3%). Analgesia was more likely to be used in brachycephalic types than in non-brachycephalic types (62.34% versus 51.43% respectively, *P* = 0.004). Analgesia usage did not differ between spaniel and non-spaniel types (*P* = 0.184).

Surgical CUD management was used in 142 (17.0%) cases. Surgery was less likely to be performed on spaniel compared with non-spaniel types (7.69% versus 18.75% respectively, *P* = 0.002). The probability of surgery did not differ between brachycephalic types and non-brachycephalic types (*P* = 0.137). Of the 834 cases overall, 62 (7.4%) were referred for advanced clinical management, the owners sought a second opinion at another primary-care practice in 13 (1.6%) cases, and 3 (0.4%) were transferred to the charity sector. There was no significant difference in the probability of referral between brachycephalic (24/239, 10.04%) and non-brachycephalic dogs (38/595, 6.39%) (*P* = 0.069)), or between spaniels (9/130, 6.92%) and non-spaniels (53/704, 7.53%) (*P* = 0.809). Dogs that were referred were more likely to receive surgical management (26/62, 41.94%) than dogs that were not referred (116/772, 15.03%) (*P* < 0.001).

At the end of the study period, the clinical records indicated CUD resolution in 658 (78.9%) cases. Brachycephaly was not associated with the probability of resolution (*P* = 0.392) but spaniels were less likely to have resolved than non-spaniels (*P* = 0.004). Resolution was less likely in dogs that were referred (67.7%) than in those that were not referred (81.0%) (*P <* 0.001). Recurrence of CUD in the same eye following resolution during the study period was recorded in 45 (5.4%) of cases. Ninety-four (11.3%) of the CUD cases died of all causes during the study period, with 74 (78.7%) of these deaths involving euthanasia. CUD was recorded as contributing to 10 (13.5%) of these 74 euthanasia decisions.

Univariable logistic regression modelling identified five variables that were liberally associated with CUD and were further evaluated in the main multivariable logistic regression modelling: *breed, bodyweight relative to breed mean, age, neuter* and *insurance*. The final main multivariable model retained three risk factors: *breed, age,* and *insurance*. No biologically significant interactions were identified in the final model. The final model showed good discrimination (area under the ROC curve: 0.7368). After accounting for the effects of the other variables evaluated, 15 breeds showed increased odds of CUD compared with crossbred dogs. The breeds with the highest odds included the Pug (OR: 19.05, 95% CI 13.45–26.97, *P* < 0.001), Boxer (OR: 12.12, 95% CI 8.77–16.76, *P* < 0.001) and Shih Tzu (OR: 10.04, 95% CI 7.30–13.80, *P* < 0.001). Compared with dogs aged < 3.0 years, dogs aged 6.0–8.9 years had 2.24 times the odds (95% CI 1.82–2.75, *P* < 0.001) of CUD. Insured dogs had 1.6 (95% CI 1.33–1.92, *P* < 0.001) times the odds of CUD compared with uninsured dogs (Table 3).

**Table 3:** Final multivariable logistic regression results

Variable	Category	Odds ratio	95% CI^a^	*P*-value
Common breeds	Crossbreed	Base		
	Pug	19.05	13.45–26.97	< 0.001
	Boxer	12.12	8.77–16.76	< 0.001
	Shih Tzu	10.04	7.30–13.80	< 0.001
	Cavalier King Charles Spaniel	6.10	4.36–8.54	< 0.001
	Bulldog	7.76	4.69–12.84	< 0.001
	King Charles Spaniel	5.62	2.98–10.62	< 0.001
	Lhasa Apso	4.97	3.01–8.22	< 0.001
	French Bulldog	7.25	3.92–13.42	< 0.001
	West Highland White Terrier	2.05	1.34–3.15	0.001
	Staffordshire Bull Terrier	2.50	1.81–3.45	< 0.001
	Cocker Spaniel	2.14	1.45–3.15	< 0.001
	English Springer Spaniel	1.93	1.17–3.19	0.010
	Border Terrier	2.00	1.06–3.75	0.032
	Chihuahua	2.54	1.50–4.28	< 0.001
	Yorkshire Terrier	1.78	1.13–2.80	0.013
	Jack Russell Terrier	1.45	1.00–2.11	0.050
	Border Collie	0.96	0.53–1.77	0.907
	Labrador Retriever	0.57	0.36–0.90	0.016
	Other purebreds	1.30	1.00–1.69	0.052
Age category (years) (pre-existing cases included as non-recorded)	< 3.0	Base		
	3.0–5.9	0.96	0.76–1.21	0.724
	6.0–8.9	2.24	1.82–2.75	< 0.001
	9.0–11.9	2.78	2.23–3.47	< 0.001
	≥ 12.0	2.74	2.13–3.53	< 0.001
	Not recorded	~		
Insurance	Non-insured	Base		
	Insured	1.6	1.33–1.92	< 0.001
	Not recorded	1.27	1.06–1.54	0.012

Final multivariable logistic regression model for risk factors associated with diagnosis of corneal ulcerative disease in dogs attending primary-care veterinary practices in England

^a^CI confidence interval

As described in the methods, four variables (*purebred, spaniel-brachycephalic*, *Kennel Club breed group* and *adult bodyweight*) individually replaced the *breed* variable in the final multivariable model. Purebred dogs were strongly associated with CUD, showing 2.23 times the odds (95% CI 1.84–2.87, *P* < 0.001) compared with crossbred dogs. In support of the study hypothesis, brachycephalic type had 11.18 (95% CI 8.72–14.32, *P* < 0.001) times the odds and spaniel type had 3.12 (95% CI 2.37–4.10, *P* < 0.001) times the odds compared with crossbred dogs. Four of the seven Kennel Club breed groups showed higher odds of CUD compared with dogs of breeds that are not recognized by the Kennel Club: Utility, Working, Toy and Terrier. The odds of CUD decreased as adult bodyweight increased (Table 4).

**Table 4:** Multivariable logistic regression results for variables that replaced breed

Variable	Category	Odds ratio	95% CI^a^	*P*-value
Purebred status	Crossbred	Base		
	Purebred	2.23	1.84–2.87	< 0.001
Spaniel-brachycephalic	Crossbred	Base		
	Spaniel	3.13	2.38–4.12	< 0.001
	Brachycephalic	11.18	8.72–14.32	< 0.001
	Purebred other	1.42	1.12–1.79	0.003
Kennel Club Breed Groups	Breed not Kennel Club recognised	Base		
	Toy	3.52	2.82–4.39	< 0.001
	Utility	3.99	3.16–5.03	< 0.001
	Terrier	1.83	1.44–2.35	< 0.001
	Gundog	0.97	0.75–1.25	0.811
	Hound	1.04	0.66–1.64	0.860
	Pastoral	0.83	0.56–1.22	0.343
	Working	3.64	2.76–4.79	< 0.001
Adult (> 9 months) bodyweight (kg)	< 10.0	1.88	1.47–2.41	< 0.001
	10.0–19.9	1.70	1.33–2.18	< 0.001
	20.0–29.9	1.26	0.96–1.64	0.090
	30.0–39.9	Base		
	≥ 40.0	0.69	0.45–1.05	0.086
	Not available	0.73	0.53–1.02	0.064

Results for variables that replaced the breed variable in the final multivariable logistic regression model (with age category and insurance status) to evaluate risk factors associated with a diagnosis of corneal ulcerative disease in dogs attending primary-care veterinary practices in England

^a^CI confidence interval

## Discussion

This is the first study to explore the wider presentation of CUD in dogs attending primary-care practices by analysing clinical data from a multicentre primary-care research database. Previous studies have mainly relied on referral populations [5–10]. This current study design aimed to explore primary-care clinical data in order to reduce selection bias issues that frequently limit the generalisabilty of research based on referral caseloads [11]. It is also the first study to confirm brachycephalic and spaniel breed types as major risk factors for the diagnosis of CUD in general practice caseloads. This is useful evidence for ophthalmologists to support their advice to referring general practices as well as for primary-care veterinarians tasked with diagnosing and managing dogs with CUD.

The power of research to report precise prevalence estimates and to explore multiple risk factors is dependent on large study sample sizes [47]. The case counts used in previous publications on CUD have generally been small [5, 7–10, 16–29]. Although the overall study population of some of these earlier studies may have included up to 200 animals [5, 21, 26], the count of CUD cases within each population, when clearly presented, often dropped to much lower numbers [5]. Furthermore, many of these earlier studies only included referral caseloads, which introduces inevitable selection bias into the study design [11]. In contrast, the present study analysed data from 104,233 dogs attending primary-care practices, among which there were 834 patients recorded with CUD. This, to the best of the authors’ knowledge, makes the present study the largest of its kind, and the only one based on an exclusively primary-care practice population, and therefore offers a CUD clinical perspective that has previously not been reported [12, 48].

The results of the present study show strong evidence through the analysis of large numbers that purebred dogs in the general population are more likely to be diagnosed with CUD than non-purebred dogs (2.23 times the odds), and that brachycephalic (11.18 times the odds) and spaniel types (3.12 times the odds) in particular appear especially predisposed to diagnosis of CUD. Previous studies on smaller, referral populations have also suggested that brachycephalic breeds and those non-brachycephalic breeds that also have prominent eyes could be overrepresented for CUD [7, 9, 31, 49]. Brachycephalic conformation has been associated with reduced numbers of corneal nerve endings [50]. Studies have also shown that corneal sensation as measured by corneal aesthesiometry is reduced in brachycephalic dogs and cats [50, 51]. It is also known that denervation of the cornea can lead to pathology of the corneal epithelium [52–55], and that corneal limbal stem cell niches require a nurturing relationship with corneal innervation [56]. Therefore, reduced corneal sensation and fewer numbers of corneal nerves that are physiologically associated with the brachycephalic skull conformation may lead to an increase in, or at least predispose to, the development of CUD, and might at least partially explain why brachycephalic breeds were more likely to be diagnosed with CUD in the present study. However, other common periocular features of brachycephalic dogs, such as the presence of a large palpebral aperture and a shallow socket, may additionally play interactive roles in the pathogenesis of CUD by promoting greater exposure of the less sensitive cornea [31, 57, 58]. It is interesting to note that in the current study, spaniels also had an increased risk of CUD. Morphologically, some spaniels have a relatively large interpalpebral fissure and shallow socket when compared to non-spaniel and non-brachycephalic breeds, and it is possible this conformation might play a role in development of CUD in spaniel breeds too [31]. Because the precise factors that promote CUD in brachycephalic and spaniel breeds remains speculative, these aspects warrant further study and may offer possible breeding selection routes towards reducing the impact of CUD on the welfare of these breed types.

The current study showed that compared with crossbred dogs, the Pug, Boxer and Shih Tzu showed very high breed predilections for CUD. It is notable that these predisposed breeds were all brachycephalic with worryingly high results and therefore a major group effort from ophthalmologists, primary-care veterinarians, researchers, owners and breeders is urgently needed to reduce CUD in these highly-predisposed breeds. A previous study on the overall disorder burden of Pugs reported corneal disorders as the second most common individual condition of Pugs (8.72% prevalence) and identified ophthalmological disorders as the most common group of disorders in Pugs overall (16.25% prevalence) [59].

Adverse welfare effects are an important aspect of CUD in dogs from a perspective of presence of pain and mortality [3]. Retention of vision and loss of the eye may also be considered as long-term welfare issues for affected individuals although we do not currently have the objective data on which to base definitive conclusions on these judgements [4]. It is important to check central corneal clarity following CUD management as this impacts on long-term vision [60]. A validated corneal clarity scoring system for veterinary cases exists and its use, as well as the recording of the results obtained into the clinical notes, should be encouraged in all cases of CUD [60]. The present study did not aim to carry out an in-depth analysis of primary-care diagnostics and management for CUD. However, it did aim to extract data on basic clinical management that could assist with welfare evaluations and that could be complemented by future more-focused studies that specifically aim to increase our understanding of primary-care CUD management. Keratoconjunctivitis sicca has been associated with the development of CUD, especially in brachycephalics, and it is recommended that dogs with corneal ulcers undergo tear measurement with Schirmer tear test-1 [7]. In the present study, just 23.7% of CUD cases underwent Schirmer tear testing.

Evaluation of welfare impact for disorders at a population level is important to assist with evidence-based ranking of disorders and strategising the distribution of limited resources for welfare improvement, for example through regulatory or legislative change, genetic advances or breeding schemes [61]. A GISID (generic illness and severity index for dogs) has been proposed that aims to objectively generate welfare risk scores using data on prevalence, duration and severity [61]. The current study contributes some information that may assist efforts to evaluate and rank CUD within the overall spectrum of disorders in dogs. Overall, 69.1% of CUD cases had either pain recorded in their notes and/or received pain management, suggesting that the attending veterinary surgeons perceived CUD to be a painful condition in a high proportion of cases. The use of surgical management in 17.0% of the CUD cases in the current study may have also introduced an element of surgical pain for these cases but successful surgery may also have hastened recovery and therefore reduced the overall pain experienced for many dogs [62]. The current study also recorded a recurrence rate of 5.4% although the follow-up time was limited and a longer study may lead to increases in this result. CUD also contributed the decision-making for 10 of the 74 (13.5%) dogs that were euthanased during the study. However, interpretation of ocular pain findings must be taken with great care. Despite the existence of studies on the validation of systemic pain scores for use in companion animals [63–65], there are no scoring systems validated for ocular pain in veterinary species and ocular pain appreciation remains subjective. It is clear, that there is a great need for veterinary professionals to develop a reliable tool to assess ocular pain, which is currently lacking. However, it should be noted that the current study was primarily an epidemiological investigation and therefore cannot fully address the impacts of CUD on the affective state of CUD cases. This would require a multifaceted focus on physiological, behavioural, cognitive and subjective components [66]. Only 62 (7.4%) cases were referred for CUD management, which means the majority of cases were entirely under primary-care clinical management. Referral, for the most part, aims to offer ophthalmic patients and their owners the assessment and opinion of a dedicated specialist and access to on-site microsurgical facilities for corneal reconstruction if deemed necessary [67]. Despite this, it is worth noting that 81.0% of the CUD cases that were managed entirely within the primary-care setting had resolved by the end of the study. As mentioned earlier, however, assessment of treatment success of CUD should also include the corneal clarity score achieved after treatment [60]. Although non-referral might appear unrelated to a positive outcome in this study, saving an eye alone does not necessarily equate to treatment success if vision is lost as a result of CUD.

Lastly, it was noted that insured dogs in the current study had 1.6 times the odds of CUD diagnosis compared with non-insured dogs. This tendency towards enhanced diagnostic probability in insured animals has now been demonstrated across a wide range of disorders and there is a trend for the diagnostic impact from pet insurance to increase as disorders require more expensive or complicated diagnostic protocols [36, 68–71]. Pet insurance may reduce financial constraints for both the owner and the veterinarian and consequently encourage earlier and more frequent veterinary visits and allow greater diagnostic freedom with consequential gains to animal welfare [72]. It is also possible that owners who purchase pet insurance might have stronger emotional bonds with their animal or higher commitment to providing the best medical therapy regardless of any financial constraints [73].

The study had some limitations. As previously described, these data were not recorded primarily for research purposes and thus were limited by some missing data as well as reliance on accurate and thorough record-keeping of the clinicians [69, 71, 74]. The study included all cases diagnosed with CUD and did not attempt to categorize these based on etiological subsets. The current study defined specific lists of breeds that were described as ‘brachycephalic’ and ‘spaniel’ types and aimed for an overall exploration of associations between these types of dogs with CUD. Explanations for the specific selections are included in the methods section but reasoned arguments could equally be made to extend these lists to include other breeds or for the removal of certain breeds from the current lists. The authors accept that changing the breed listings may impact somewhat on the specific results but is unlikely to substantially change the overall inference. Brachycephaly is not a fixed or binary conformational attribute but exists on a continuum from extreme to moderate brachycephalism that can be scored more precisely by newer cephalic index systems derived from various skull width to skull length ratios [44] Additionally, it is worth noting that, not all individuals may necessarily even be brachycephalic among breeds that are traditionally considered as a typically brachycephalic type. Inference was drawn on the opinion of the attending practitioners on the painfulness of CUD by extracting data on whether ‘pain’ was recorded in the clinical notes and whether analgesia was administered. However, neither of these actions are necessarily proof of true pain and, conversely, lack of recording of pain or failure to use analgesics do not necessarily preclude the presence of pain.

## Conclusions

This is the first study to explore the prevalence and odds of CUD based on skull conformation and breed in a large general population of dogs attending primary-care practice. The results provide evidence for the first time of a strong predisposition to CUD in brachycephalic and spaniel breed types in the general population. The results and conclusions presented here can assist ophthalmologists, general practitioners, breeders and owners by improving breed-based advice about CUD diagnosis, management and prevention.

## Abbreviations

CI: Confidence intervals
CUD: Corneal ulcerative disease
EPR: Electronic patient record
KCS: Keratoconjunctivitis sicca
OR: Odds ratio
STT-1: Schirmer tear test-1
